# Selective anti-CXCR2 receptor blockade by AZD5069 inhibits CXCL8-mediated pro-tumorigenic activity in human thyroid cancer cells in vitro

**DOI:** 10.1007/s40618-024-02410-6

**Published:** 2024-06-20

**Authors:** F. Coperchini, A. Greco, E. Petrosino, L. Croce, M. Teliti, N. Marchesi, A. Pascale, B. Calì, P. Pignatti, F. Magri, M. Uddin, M. Rotondi

**Affiliations:** 1 Department of Internal Medicine and Therapeutics, University of Pavia, Via S. Maugeri 4, 27100 Pavia, Italy; 2 Unit of Endocrinology and Metabolism, Laboratory for Endocrine Disruptors, Istituti Clinici Scientifici Maugeri IRCCS, 27100 Pavia, Italy; 3 Unit of Pharmacology, Department of Drug Sciences, University of Pavia, 27100 Pavia, Italy; 4 Department of General and Minimally Invasive Surgery, Istituti Clinici Scientifici Maugeri IRCCS, 27100 Pavia (PV), Italy; 5 Allergy and Immunology Unit, Istituti Clinici Scientifici Maugeri IRCCS, 27100 Pavia, Italy; 6 AstraZeneca Gothenburg, Biopharmaceuticals R&D, Mӧlndal, Sweden

**Keywords:** AZD5069, Thyroid cancer, CXCL8, CXCR2 receptor

## Abstract

**Background:**

Thyroid cancer is the most common endocrine malignancy. Current therapies are successful, however some patients progress to therapeutically refractive disease. The immunotherapeutic potential of the CXCL8-chemokine/CXCR2-chemokine-receptor system is currently being explored in numerous human cancers. This study aimed to evaluate if the targeting of CXCR2 by its selective antagonist, AZD5069, could modulate CXCL8-mediated pro-tumorigenic effects in thyroid-cancer (TC) cells in vitro.

**Methods:**

Normal human primary thyroid cells (NHT) and TC cell lines TPC-1 (RET/PTC), BCPAP, 8505C and 8305C (BRAFV600e) were treated with AZD5069 (100 pM-10 µM) over a time-course. Viability and proliferation were assessed by WST-1 and crystal violet assays. CXCL8 and CXCR2 mRNA were evaluated by RT-PCR. CXCL8-protein concentrations were measured in cell culture supernatants by ELISA. CXCR2 on cell surface was evaluated by flow-cytometry. Cell-migration was assessed by trans-well-migration chamber-system.

**Results:**

AZD5069 exerted negligible effects on cell proliferation or viability. AZD5069 significantly reduced CXCR2, (but not CXCL8) mRNAs in all cell types. CXCR2 was reduced on the membrane of some TC cell lines. A significant reduction of the CXCL8 secretion was found in TPC-1 cells (basal-secretion) and NHT (TNFα-induced secretion). AZD5069 significantly reduced basal and CXCL8-induced migration in NHT and different TC cells.

**Conclusions:**

Our findings confirm the involvement of the CXCL8/CXCR2-axis in promoting pro-tumorigenic effects in TC cells, further demonstrating its immunotherapeutic significance in human cancer.

**Supplementary Information:**

The online version contains supplementary material available at 10.1007/s40618-024-02410-6.

## Introduction

Thyroid cancer (TC) is the endocrine cancer with the highest prevalence and incidence worldwide [1, 2], being papillary thyroid carcinoma the most frequent subtype in histology [3]. The incidence of TC has markedly increased globally in recent years [1, 4], with an estimated new diagnosed cases of around 449,000 in women and 137,000 in men in 2020 [5]. The main strategies for effective treatment of TC include thyroidectomy, followed by radioiodine treatment and Thyroid-stimulating hormone (TSH) suppressive therapy in selected patients [6]. However, there is still a subset (≈ 3–5%) of patients that progress to therapeutically refractive disease, constituting the so-called “TC-related deaths due to the lack of effective treatment”. For this reason, a greater mechanistic understanding of the pro-tumorigenic pathways involved is needed to develop personalized therapies for these patients [7]. The tumor microenvironment (TM) largely influences TC biological behaviour by modulating both the proliferation and metastatic potential of cancer cells [8]. The balance among the components of the thyroid cancer microenvironment (TCM), drives malignancy progression and, ultimately influences the outcome of patients [9, 10].

TCM is composed of both solid (cell features like cancerous, normal and immune infiltrate) and soluble mediators (chemokines, cytokines and growth factors), among which Interleukin-8 (named also CXCL8 or IL-8), is the chemokine most widely investigated not only in TC but also in several types of human cancers [11, 12]. CXCL8 is a pro-tumorigenic CXC chemokine exerting pro-angiogenetic and proliferative effects. CXCL8 is a major mediator of immune cell trafficking, including neutrophils via expression of the G-protein-coupled receptors, C-X-C motif chemokine receptor 1 (CXCR1) and C-X-C motif chemokine receptor (CXCR2), within the TCM.

CXCL8 also plays a crucial role in the process of epithelial to mesenchymal transition (EMT), consequently promoting thyroid cancer cell migration to metastatic sites [11]. It was recently shown that the inhibition of the secretion of CXCL8 by different pharmacological compounds reduces thyroid cancer cell migration in vitro [11]. In addition, data on TC pre-clinical models found that treatment with rh-IL-8 significantly increased the metastatic spread, whereas targeting of IL-8 by a neutralizing monoclonal antibody (mAb) prolonged mice survival [13]. Concerning in vivo data, IL-8 levels were associated with the aggressiveness of several types of cancer, including the thyroid phenotype [14]. AZD5069 (developed by AstraZeneca) is a small molecule antagonist reported to have > 100-fold selectivity for CXCR2 over the CXCR1 receptor, that is well tolerated without adversely affecting neutrophil-mediated host immunity [15, 16]. Treatment with AZD5069 was shown to inhibit the recruitment of tumour-associated neutrophils (TANs) into peritumoral regions in vivo [17]. Furthermore, in patients with advanced metastatic tumours, treatment with an anti-IL-8 mAb such as BMS-986253 s (NCT02536469), plus an anti-PDL-1 induced a significant reduction of CXCL8 serum levels and increased anti-tumor activity (NCT03400332). Recent data showed that the CXCR2 receptors are particularly involved in tumour progression. Evidence indicates that: (i) CXCR2 deletion reduces the metastatic potential of lung cancer cells [18]; (ii) in breast cancer models, CXCR2 signaling is important for attracting myeloid-derived-suppressor-cells to the TM, where they drive invasion and metastasis [19]; (iii) CXCR2 inhibition enhances T cell entry conferring sensitivity to anti-PD1 therapy in pancreatic cancer [20]; (iv) CXCR2 is a very important target for suppressing neutrophilic inflammation in breast cancer models [21]. Regarding TC, few data on chemokine-receptor-related immunotherapy are available [22]. Treatment with AZD5069 was shown to inhibit neutrophil recruitment to breast cancer brain metastasis [23]. In addition, Phase I/II trials with AZD5069, administered in combination with enzalutamide are ongoing in patients with metastatic castration-resistant prostate cancer (NCT03177187). Furthermore, an ongoing clinical trial is evaluating AZD5069 in combination with an anti-PDL1 mAb, in patients with head and neck cancer showing metastasis and in patients with solid tumors in an advanced stage (NCT02499328).

We hypothesized that targeting thyroid cancer cells using the selective CXCR2 receptor antagonist AZD5069 may reduce the CXCL8-mediated pro-tumorigenic effects (i.e. via inhibition of cell migration) and/or suppress the tumour cell ability to secrete CXCL8 endogenously. Thus, the present in vitro study aims to investigate the therapeutic effects of AZD5069 in differentiated thyroid cancers in terms of thyroid cancer cell viability, proliferation, CXCL8 secretion and cell migration.

## Materials and methods

### Normal human thyroid cells (NHT) in primary cultures

Primary cultures were obtained from surgical specimens of human thyroids derived from patients (n = 5) undergoing thyroidectomy for multinodular goiter (n = 2) and toxic adenoma (n = 3). All patients were euthyroid, and none of them showed positive tests for thyroglobulin antibody and thyroperoxidase antibody. Primary cells are generally preferred to commercial normal thyroid cell lines because available normal thyroid cell lines do not completely reflect the phenotype of normal thyrocytes. The study was approved by the Institutional Board of ICS-Maugeri, Pavia, Italy. Signed informed consent was obtained from all patients. All the experiments were performed according to the relevant guidelines and regulations and adhered to the principles of the Declaration of Helsinki. Primary cultures were obtained based on the following protocol: (i) shredding of tissues, (ii) incubation in collagenase ( type II, Sigma, Saint Louis, MO, USA) for 4 h at 37 °C as previously described [24], (iii) filtering of the obtained homogenate, (iv) spun at 1000×*g* for 10 min for two times washing with Coon’s F12 medium, (v) resuspend in Coon’s & H medium (composed by 5% newborn calf serum, five hormones and bovine TSH (1 mU/ml) [24].

### Cultures of thyroid tumour cell lines

The human thyroid cancer cell lines used in this study are BCPAP, 8505C, 8305C (BRAF V600E), and TPC-1 (RET/PTC). The DNA analysis was performed to authenticate these cells.

Dulbecco’s Modified Eagle Medium (DMEM) (Sigma, Saint Louis, MO, USA) was supplemented with required components as previously described and used for BCPAP and TPC-1, whereas 8303C cells were propagated in MEM, and 8505C in RPMI supplemented with appropriate adjunctive components as previously described [24]. The incubation of cells with the selected stimuli was performed in a serum-free medium.

### WST-1 for the viability assay

Cells were grown in complete medium until reaching 80% confluence, then, were harvested and seeded in 96-well flat plates (2 × 10^4^ cells/well). The complete medium was supplemented with increasing concentrations of AZD5069 (100 pM–10 µM, kindly provided by AstraZeneca), the concentrations of AZD5069 were chosen based on previous in vitro studies [23, 25] and according to those reached in the sera of patients treated with this compound [26]. Cells were incubated at three different time points 24, 48 and 72 h. At the end of each time point, cells were incubated with WST-1 in a cell cultures incubator. After 30 min the absorbance was measured with a microplate plate reader (450 nm, Victor NIVO Multimode Plate Reader, PerkinElmer). All experiments were performed in triplicates.

### Cell proliferation assay

NHT and human thyroid cancer cell lines were seeded in a 96-well plate at a density of 3000 cells per well and incubated in the presence or absence of increasing concentrations of the selective CXCR2 antagonist, AZD5069 (100 pM–10 µM) for 24, 48, 72 and 144 h. Cells were fixed with methanol for 20 min and stained with 0.5% crystal violet dye (C0775; Sigma-Aldrich) for 5 min [27]. Cell proliferation was evaluated under an inverted microscope Olympus BX51 (Olympus, Deutschland GmbH, Hamburg, Germany). 1% sodium dodecyl sulfate SDS was added to cells for induce the release of (436143; Sigma-Aldrich) crystal violet dye. The optical density (OD) was measured at 570 nm. All experiments were performed in triplicates.

### Basal and tumor-necrosis-factor-α (TNF-α) -induced CXCL8 secretion in thyroid *cancer* cells in the presence or absence AZD5069

3000 cells were seeded into 96-well plates. After 24 h cells were incubated in serum-free medium alone (basal condition) or supplemented with AZD5069 (100 pM–10 µM). This treatment was performed in the absence (basal secretion) and with (stimulated condition) of TNF-α (10 ng/ml). Experiments were performed in triplicates.

### Enzyme-linked immunosorbent assay (ELISA)

In thyroid cultures cell supernatants CXCL8 levels were measured using ELISA kits (R&D Systems, Minneapolis, MN). Samples were assayed in duplicates.

### Real-time (RT) polymerase-chain-reaction (PCR)

RNA was obtained from BCPAP, TPC-1, 8305C, 8505C and NHT incubated to AZD5069 1 µM for 24 h. Total RNA purification kit (Norgen Biotek, Canada) was used to obtain RNA from thyroid cells) cDNA was then reverse transcribed by a SensiFast c-DNA synthesis kit (Bioline, London, UK). Real-time PCR was performed using Sensi-Fast SYBR Green Hi-ROX kit (Bioline, London, UK) on StepOne Plus Applied Biosystems real-time PCR system. Pre-designed primers targeting CXCL8 (F: CAGTGCATAAATACTC; R: CTCTTCAAAACTCCAC) and CXCR2 (F: ATTCTGGGCATCACAG; R: TGCACTTAGGAGGTCT) were obtained from Biomers.net GMBH (Soflinger, Germany). Primers were chosen based on previously published data [28]. GAPDH was used as endogenous control. GAPDH (F: AAATCCCATCACCATCTTCC; R: GGTTCACACCCATGACGAAC). Results were normalized for GAPDH and analyzed using the ΔΔCt method.

### Flow cytometric evaluation of CXCR2 expression in NHT cells, TPC-1, BCPAP, 8505C and 8305C cell lines after treatment with AZD5069

NHT, TPC-1, BCPAP, 8505C and 8305C cells were seeded in a 6 well plate at 3 × 10^4^ cells concentration per well. Cells were incubated for 24 h with complete medium alone (basal condition) or with AZD5069 1 µM. At the end of the incubation, the culture supernatant was removed and cells were detached from dishes using a 0.5 mmol/L EDTA solution (Sigma, Saint Louis, MO, USA). 3 × 10^4^ cells were incubated for 30 min (on ice and in the dark) with phycoerythrin (PE)-labelled mouse anti-human CXCR2 antibody (clone 6C6, BD Pharmingen, BD Biosciences, San Jose, CA) or with a control isotype antibody IgG1κ (PE, BD Pharmingen). Cells were washed with phosphate-buffered saline/bovine serum albumin (PBS/BSA 0.5%) and acquired with a flow cytometer (FACScan Becton Dickinson). FACS analysis was performed using the CellQuest software (BD Biosciences).

Data are expressed as percentage of CXCR2+ cells (number of cells and mean of fluorescence intensity MFI) after treatment with AZD5069 vs. basal condition (estimated to be 100%).

### Migration

Trans-well migration chamber system was employed for these assays (Merck Millipore, Milan, Italy). Briefly, NHT and cancer thyroid cell lines were cultured with AZD5069 1 µM alone or in combination with CXCL8 (100 ng/ml) (R&D System). At the end of the incubation period, cells were seeded in the upper chambers (50 × 10^3^ cells/well) of the trans-well system (0.3 cm^2^/well membrane area and 8 μm pore size). The lower chambers were filled with cell culture medium. During 16 h at 37 °C and 5% CO_2_, cells were left to migrate. Samples were analyzed as previously described [29], at the end of the incubation by cutting inserts membrane and staining them with Hoechst 33342 (1:1000) (Life Technologies, Monza, Italy). For each condition, three replicates were evaluated. The acquisition of images was performed with an Olympus BX51 microscope (Olympus, Deutschland GmbH, Hamburg, Germany). Twelve random fields per condition were analyzed. Data are shown as the mean numbers of cells/field migrated ± standard deviation (SD).

### Statistical analysis

SPSS software was employed (SPSS, Inc., Evanston, IL). Values are reported as mean ± SD unless otherwise noted. One-way ANOVA for normally distributed variables was used for comparing mean group values. For multiple comparisons, post-hoc analysis by Bonferroni’s correction was applied. Means of Student’s t-test were used for testing between-group comparisons. Between-group comparisons were performed using Student’s t-test for unpaired data. The statistical significance was considered for *p* values < 0.05.

## Results

### Effect of a selective CXCR2 antagonist (AZD5069) on normal and tumor thyroid cells viability

**Fig. 1:**
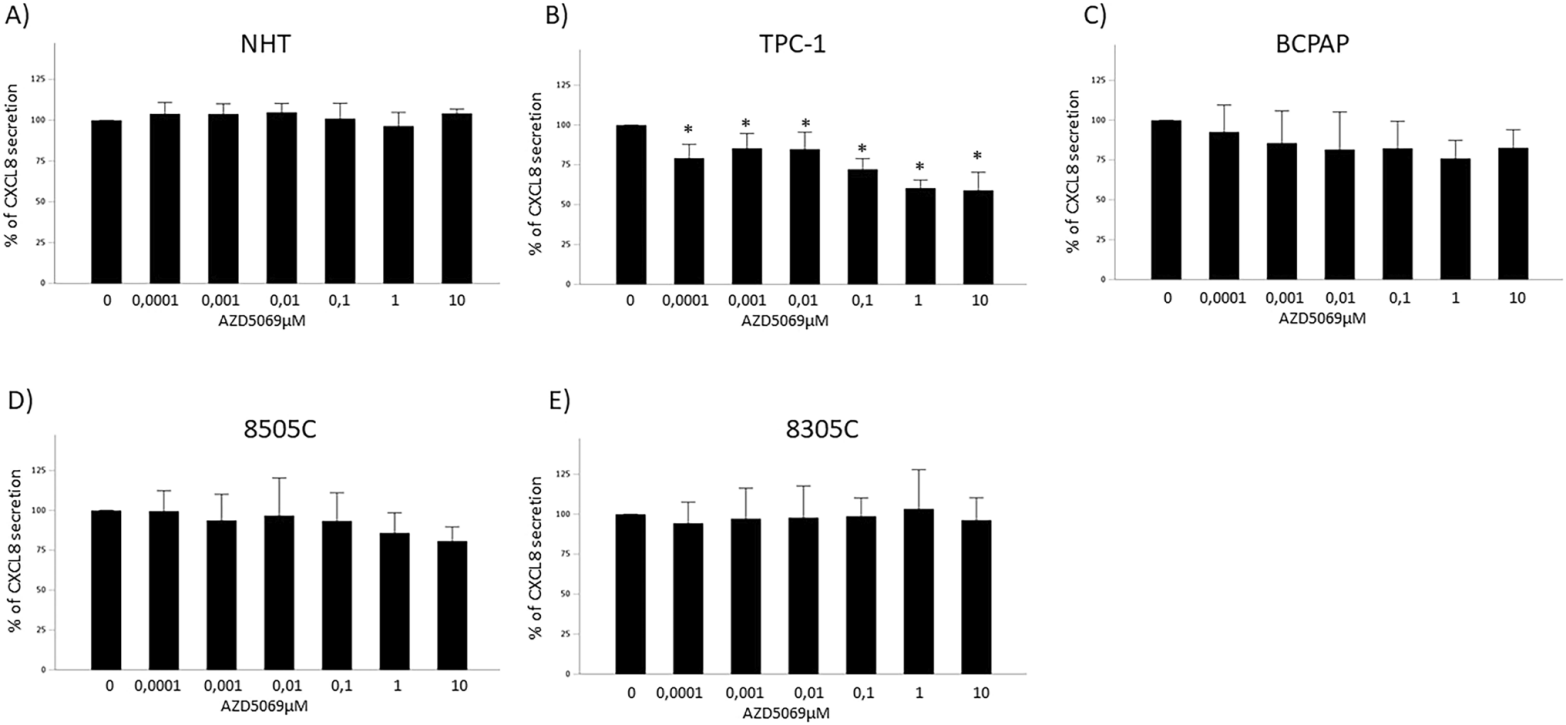
Effect of AZD5069 on basal CXCL8 secretion by normal and tumor thyroid cells. Treatment of various thyroid cancer cell lines with increasing concentrations of AZD5069 (100 pM–10 µM) indicated that the basal secretion of CXCL8 in **A** NHT cells was unaffected (ANOVA: F = 2.128, p = 0.063); **B** TPC-1 cells was significantly reduced (ANOVA: F = 28.430, p < 0.001), **C** BCPAP cells was unaffected (ANOVA: F = 1.748, p = 0.134); **D** 8505C cells was unaffected (ANOVA F = 2.105, p = 0.067); **E** 8305C cells was unaffected (ANOVA: F = 0.248, p = 0.958). *Post hoc by Bonferroni p < 0.05

To evaluate if the treatment with AZD5069 could exert any effect on cell viability, a WST-1 assay was employed. Normal human thyroid cells (NHT) and thyroid cancer cell lines (TPC-1, BCPAP, 8505C and 8305C) were treated with increasing concentrations of AZD5069 over a defined time course (24, 48 and 72 h). The results showed that AZD5069 did not affect the viability of NHT after 24 (ANOVA: F = 0.866 p = 0.513) and 48 h (ANOVA: F = 0.487 p = 0.819). However, a reduction of NHT viability was observed after 72 h, only at the highest concentration (10 µM) of AZD5069 (ANOVA: F = 5.331 p < 0.001) (Fig. 1 supplemental materials, panel A). Notably, no modification of cell viability was observed following treatment with AZD5069 in any of the thyroid cancer cell lines (Fig. 1 supplemental materials panels C, D, E, F).

### AZD5069 does not modify the proliferation of thyroid *cancer* cell lines

**Fig. 2:**
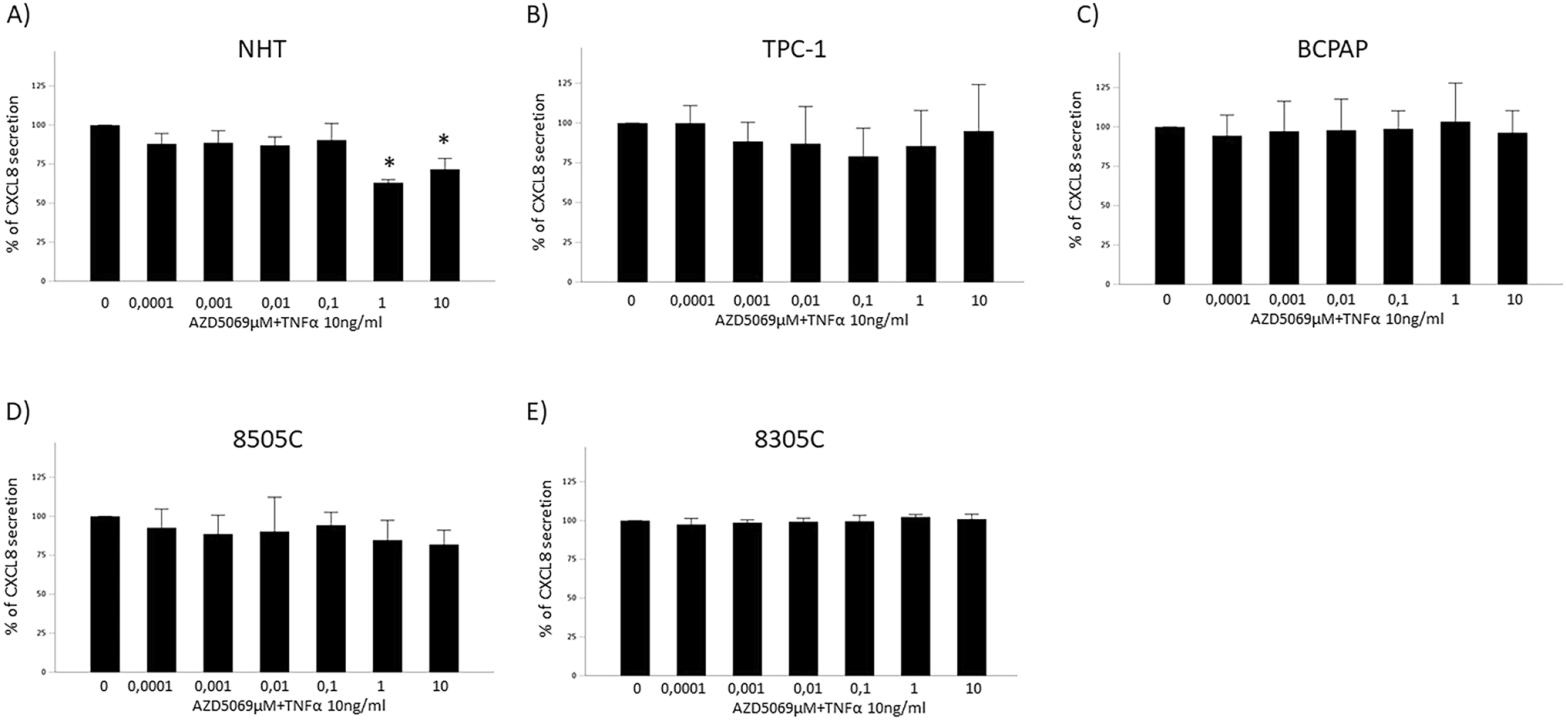
Effect of AZD5069 on the TNFα-induced CXCL8 secretion by normal and tumor thyroid cells. Treatment of various thyroid cancer cell lines with increasing concentrations of AZD5069 (100 pM–10 µM) showed that TNFα-induced CXCL8 secretion in **A** NHT cells was significantly reduced in response to the highest concentrations of the CXCR2 antagonist (ANOVA: F = 21.851, p < 0.001); **B** TPC-1 cells was unaffected (ANOVA: F = 1.913; p = 0.091); **C** BCPAP cells was unaffected (ANOVA: F = 1.912, p = 0.098); **D** 8505C cells was was unaffected (ANOVA F = 1.751,p = 0.138); **E** 8305C was unaffected (ANOVA: F = 1.997, p = 0.093). *Post hoc by Bonferroni p < 0.05

The potential ability of AZD5069 to affect the proliferation of NHT, TPC-1, BCPAP, 8505C and 8305C was tested. Briefly, thyroid cancer cells were treated with increasing concentrations of AZD5069 at 4 different time points (24, 48, 72 and 144 h). No modification of cell proliferation was observed in NHT or any thyroid cancer cell lines at any time point (Fig. 2 supplemental materials).

### The effect of AZD5069 on basal and TNFα-induced CXCL8 secretion by normal and tumor thyroid cells

To assess if AZD5069 could modulate any change in the secretion of CXCL8 by normal and tumor thyroid cells, CXCL8 concentrations were measured in cell cultures supernatants after 24 h treatment with increasing concentrations of AZD5069. AZD5069 treatment was performed in presence or absence of TNFα (10 ng/ml), a powerful inducer of CXCL8 secretion in thyroid cells [30]. Results showed that treatment with AZD5069 did not reduce the basal secretion of CXCL8 by NHT (ANOVA: F = 2.128, p = 0.063), BCPAP (ANOVA: F = 1.748, p = 0.134), 8505C (ANOVA F = 2.105, p = 0.067) or 8305C (ANOVA: F = 0.248, p = 0.958) (Fig. 1A, C, D, E).

Of note, the basal secretion of CXCL8 was significantly reduced by treatment with AZD5069 only in TPC-1 cells (ANOVA: F = 28.430, p < 0.001) (Fig. 1B).

In contrast to the observations in basal condition: (1) the TNFα-induced CXCL8 secretion was significantly suppressed by treatment with AZD5069 in NHT, even if only at the highest concentrations of the compound (ANOVA: F = 21.851, p < 0.001) (Fig. 2A); (2) no reduction of TNFα-induced CXCL8 secretion was found in TPC-1 after treatment with AZD5069 (ANOVA: F = 1.913; p = 0.091) (Fig. 2B).

Finally, a similar scenario to the one observed in basal condition was observed in BCPAP, 8505c, and 8305C, in which treatment with AZD5069 did not reduce the TNFα-induced CXCL8 secretion (ANOVAs: BCPAP: F = 1.912, p = 0.098; 8505C F = 1.751, p = 0.138; 8305C: F = 1.997, p = 0.093) (Fig. 2C–E).

### AZD5069 did not reduce the mRNA of CXCL8 in normal and tumour thyroid cells

**Fig. 3:**
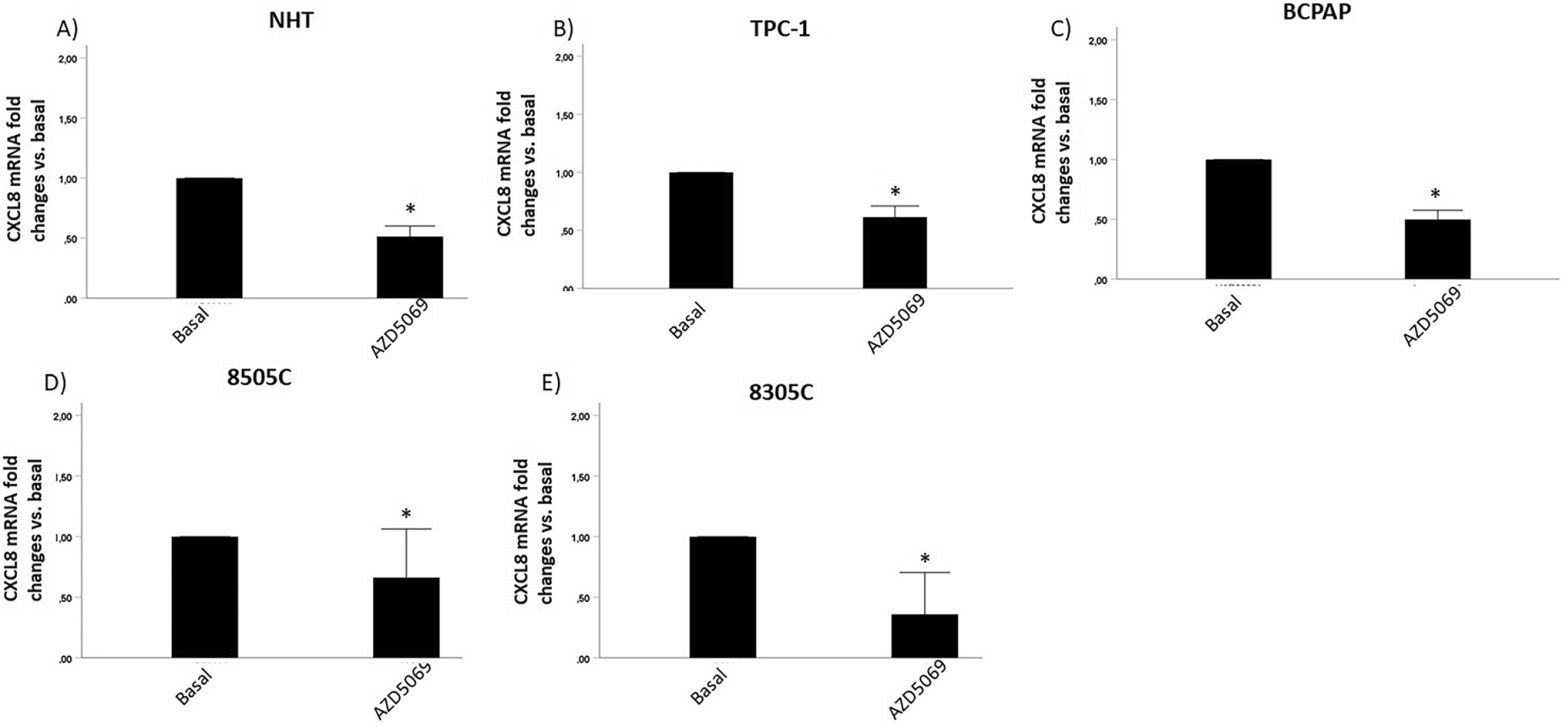
Effect of AZD5069 on CXCR2 mRNA levels in normal and tumor thyroid cells. Treatment with AZD5069 (10 Μm) significantly (Student’st-test p < 0.001) reduced mRNA levels of CXCR2 expressed by the various thyroid cancer cell lines tested: **A** NHT cells; **B** TPC-1 cells; **C** BCPAP cells; **D** 8505C cells; **E** 8305C cells

The effect of AZD5069 on CXCL8 were tested also at the mRNA level. Thyroid cells were treated with AZD5069 1 µM for 24 h, and CXCL8 mRNA levels were quantified. Results showed no modifications of CXCL8 mRNA in all thyroid cell types (both normal and cancer cell lines) treated with AZD5069 (Student’s t-test NS) (Fig. 3 supplemental materials, panels A–E).

### AZD5069 reduced the mRNA of CXCR2, the cognate CXCL8 receptor, in normal and tumour thyroid cells.

In contrast to what was observed for the ligand CXCL8, a reduction of the levels of its receptor CXCR2, was observed in both normal and tumor thyroid cells following treatment with AZD5069 [1 μM]. Indeed, as shown in Fig. 3, the mRNA levels of CXCR2 were significantly downregulated by treatment with AZD5069 in NHT, TPC-1, BCPAP, 8505C and 8305C (Fig. 3A–E) (Student’s t-test p < 0.001 for all cell types).

### Effect of AZD5069 on the cell membrane expression of CXCR2, in normal and tumor thyroid cells

**Fig. 4:**
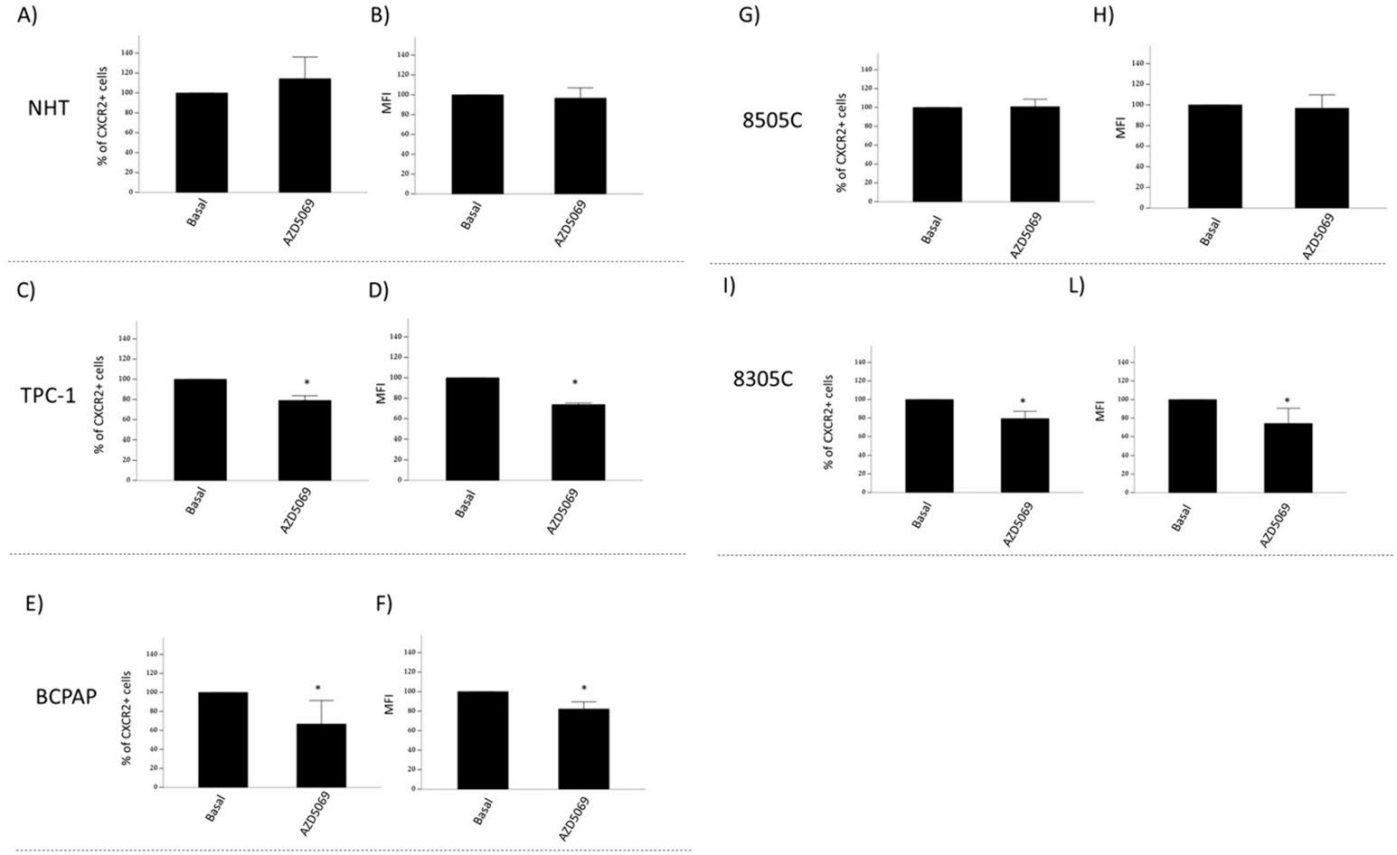
Effects of AZD5069 on CXCR2 receptor of NHT and thyroid cancer cells. Results of analysis by flow cytometer of thyroid cells treated with AZD5069 (1 μM). **A**, **C**, **E**, **G**, **I** shows the analysis of the expression of CXCR2 in terms of percentage of CXCR2+ cells in NHT, TPC-1, BCPAP, 8505C, 8305C respectively. Results showed a reduction of the percentage of CXCR2+ inTPC-1, BCPAP and 8305C (Mann Whitney test p < 0.05) but not in NHT and 8505C (Mann Whitney test NS). **B**, **D**, **F**, **H**, **L** shows the analysis of the expression of CXCR2 in terms of the amount percentage of CXCR2 recovered on cell surface (MFI) in NHT, TPC-1, BCPAP, 8505C, 8305C respectively. Results showed a reduction of the expression of CXCR2 in terms of the amount percentage of CXCR2 inTPC-1, BCPAP and 8305C (Mann Whitney test p < 0.05) but not in NHT and 8505C (Mann Whitney test NS). Data are expressed as percentage of CXCR2 + cells (number of cells and mean of fluorescence intensity MFI) after treatment with AZD5069 vs. basal condition (estimated to be 100%). *Mann Whitney test p < 0.05

CXCR2+ cells were detected by flow cytometry after a 24-h incubation with AZD5069. As shown in Fig. 4, the treatment with AZD5069 significantly reduced the percentage of the number of CXCR2 + cells in TPC-1, BCPAP and 8305C (Fig. 4C, E, I), while no changes were found in either NHT (Fig. 4A) nor 8505C (Fig. 4G) cell lines. Similar results were found when the mean of fluorescence intensity (MFI) was analyzed. The analysis of MFI led to evaluate changes in the amount of CXCR2 receptor present on the single cell membrane. Results showed that AZD5069 significantly reduced the amount of CXCR2 in TPC-1, BCPAP and 8305C (Fig. 4D, F, L), while no changes were found in NHT and 8505C cell lines (Fig. 4B–H).

### The effect of AZD5069 on the basal migration of NHT and thyroid *cancer* cell lines

**Fig. 5:**
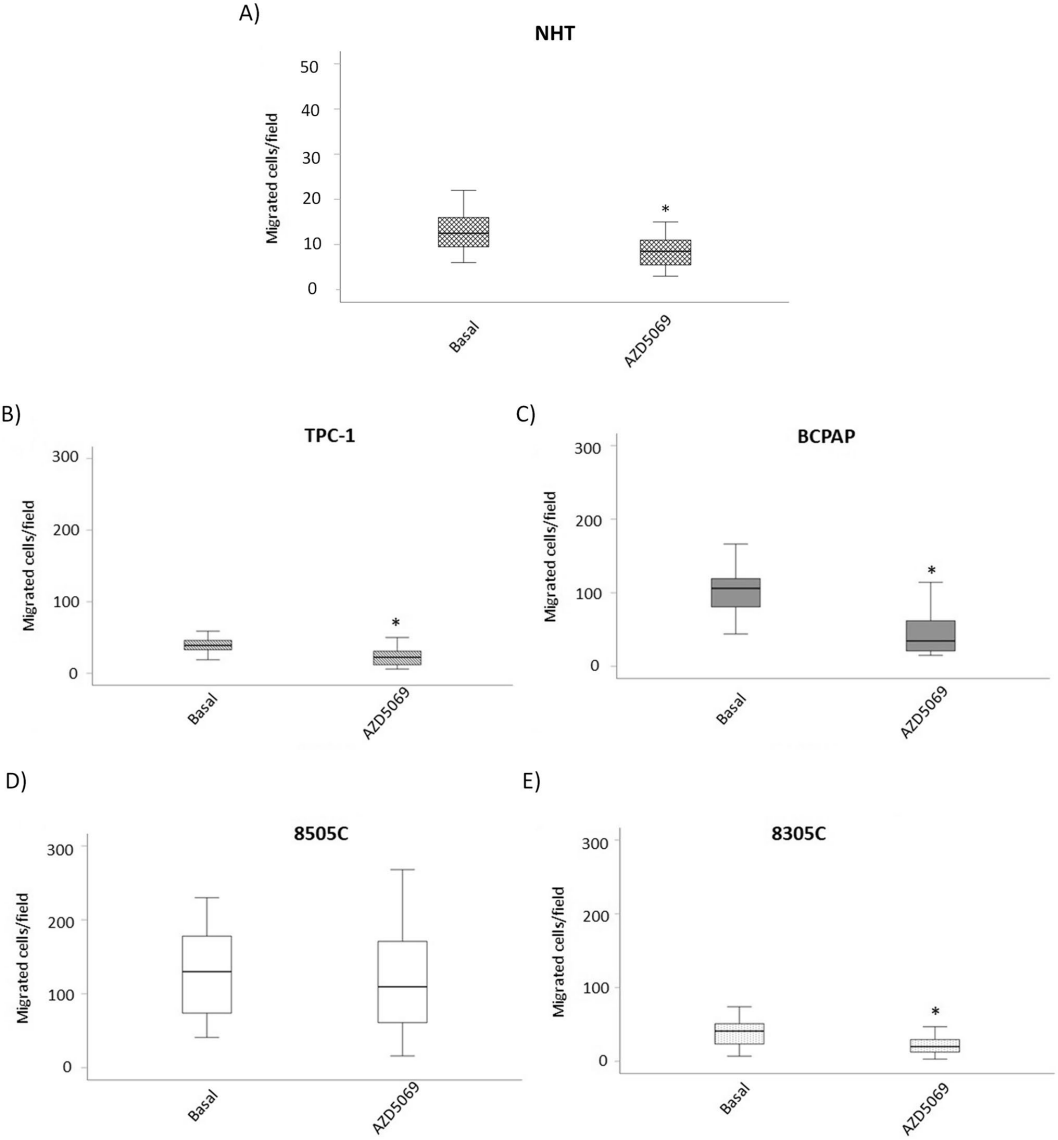
Effect of AZD5069 on the basal migration of NHT and thyroid cancer cell lines. Treatment of various thyroid cancer cell lines with AZD5069 [1 μM] showed that basal migration of **A** NHT was significantly (Mann Whitney test p < 0.001) reduced, **B** TPC-1 cells was significantly (Mann Whitney test p < 0.001) reduced, **C** BCPAP cells was significantly (Mann Whitney test p < 0.001) reduced; **D** 8505C cells were unaffected; **E** 8305C cells was significantly (Mann Whitney test p < 0.001) reduced. *Mann Whitney test p < 0.001

In order to assess if AZD5069 could reduce the basal cell migration of NHT, TPC-1, BCPAP, 8505C and 8305C, the chamber-migration assay was employed in vitro. Briefly, thyroid cancer cells were treated with 1 µM of AZD5059 for 24 h and left to migrate in the chamber-system. Results showed that treatment with AZD5069 significantly reduced, the migration of NHT, TPC-1, BCPAP and 8305C (Fig. 5A, B, C, E). On the other hand, no significant effect was observed in 8505C where the treatment with AZD5069 did not reduce thyroid cancer cell migration (Fig. 5D).

### The effect of AZD5069 on the CXCL8-induced migration of NHT and thyroid *cancer* cell lines

**Fig. 6:**
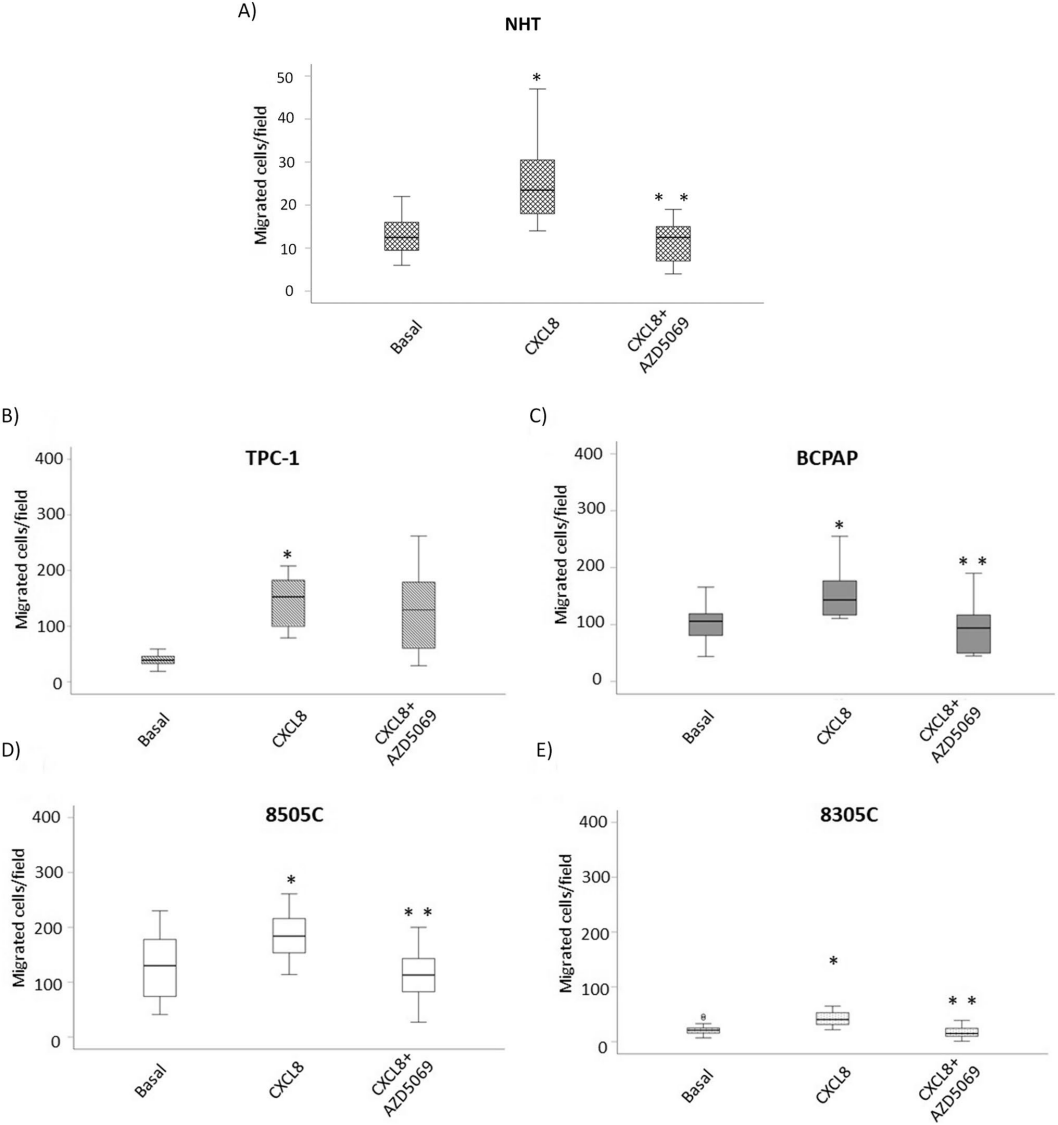
Effect of AZD5069 on the CXCL8-induced migration of NHT and thyroid cancer cell lines. Treatment of various thyroid cancer cell lines with AZD5069 (1 Μm) showed that CXCL8-induced migration of **A** NHT Kruskall Wallis; H = 38.085, p < 0.05. **B** TPC-1 cells was unaffected (Kruskall Wallis: H = 20.470 p < 0.001, post-hoc analysis by Bonferrroni NS for CXCL8 + AZD5069 *vs*. CXCL8) **C** BCPAP cells was significantly reduced (Kruskall Wallis: H = 8.213, p = 0.001), **D** 8505C cells was significantly reduced (Kruskall Wallis: H = 8.717, p < 0.01); **E** 8305C cells was significantly reduced (Kruskall Wallis: H = 29.691, p < 0.001). *Post hoc by Bonferroni p < 0.05

To assess if AZD5069 could counteract a biological pro-tumorigenic effect of CXCL8, (i.e. promotion of cell migration), in vitro migration experiments were performed in the presence of rhCXCL8 and AZD5069 alone or in combination. As expected, rhCXCL8 induced a significant migration of NHT and all thyroid cancer cell lines (TPC-1, BCPAP, 8505C and 8305C) in line with previous observations [31–33]. The concomitant treatment with AZD5069 led to a significant reduction of the CXCL8-induced cell migration in NHT (Kruskall Wallis: H = 38.085, p < 0.05), BCPAP (Kruskall Wallis: H = 8.213, p < 0,01), 8505C (Kruskall Wallis: H = 8.717, p < 0.01) and 8305C cells (Kruskall Wallis: H = 29.691, p < 0.001) (Fig. 6A, C, D, E). On the other hand the CXCL8 induced migration of TPC-1 cells was not reduced by AXZD5069 (Kruskall Wallis: H = 20.470 p < 0.001, post-hoc analysis by Bonferroni NS for CXCL8 + AZD5069 vs. CXCL8) (Fig. 6B).

## Discussion

The present study constitutes the first in vitro evaluation of the therapeutic effect of the CXCR2 receptor antagonist, AZD5069, in human normal and cancer thyroid cells. Our evaluation showed that the CXCR2 signalling pathway is a key driver of thyroid cancer cell motility and is a critical chemokine receptor targeted by AZD5069 via selective CXCR2 antagonism. Additionally, in vitro experiments enabled us to highlight the need to control endogenous CXCL8 production and consequential auto-stimulatory signalling in TC cells.

Specifically, AZD5069 was tested for its potential ability to affect: (i) thyroid cells viability and proliferation; (ii) basal levels of CXCL8 mRNA; (iii) the secretion of CXCL8 protein in both basal condition and after stimulation with TNFα); (iv) the mRNA expression levels of the receptor CXCR2; (v) basal and CXCL8-induced TC cells migration.

The results showed that AZD5069 treatment did not affect viability and proliferation of TC cells, even if a reduction of normal thyrocytes viability was observed only after incubation with the highest concentration of AZD5069 (10 µM) and for the extended duration of treatment exposure (72 h).

CXCL8 secreted by cancer cells in tumor microenvironment contributes to cancer cell proliferation, EMT and inhibition of apoptosis both in an autocrine and paracrine manner [11, 21, 34]. Moreover, CXCL8 can act in a paracrine manner to change the composition of immune infiltration in TME, by recruiting tumor-associated macrophages (TAMs), myeloid-derived suppressor cells (MDSCs), and neutrophils to the TME, resulting in dampening the anti-tumour immune response of cytotoxic immune cells [35, 36]. Based on the notion that CXCL8 secretion can also occur due to the agonism of CXCL8 with its cognate receptors CXCR2 [11, 37], we tested the hypothesis that the selective antagonism of CXCR2 by AZD5069 could suppress the autocrine stimulation of thyroid cells, resulting in reduced CXCL8 secretion. Thus, the effect of AZD5069 on CXCL8 was evaluated both at the mRNA and the protein levels. While CXCL8 mRNA expression levels were not affected by AZD5069 in any of the cancer cell types, some specific patterns of inhibition were observed for the CXCL8 protein. Indeed, in TPC-1 cells, treatment with AZD5069 elicited a significant reduction of basal CXCL8 secretion as measured in the cell supernatants, Furthermore, TNFα-induced CXCL8 secretion was significantly reduced by AZD5069 treatment only in normal thyrocytes and at the highest concentrations.

The main findings of the present study are represented by the impact on the CXCR2 mRNA and protein-membrane expression modulation and for the thyroid cancer cell migration after treatment with AZD5069. Indeed, a significant down-regulation of CXCR2 mRNA expression was observed in both normal and cancer thyroid cells after treatment with AZD5069. This finding is in line with a previous clinical study showing that treatment with AZD5069 induced downregulation of the CXCR2 receptor mRNA levels in sputum cell pellets from patients with severe asthma [25]. The results observed at mRNA levels on thyroid cells were in line with those obtained by FACS evaluation of CXCR2 protein membrane expression. Indeed AZD5069 reduced the percentage of CXCR2 positive cells as well as the amout of CXCR2 on cell surface in TPC-1, BCPAP and 8305C. The lack of reduction found in NHT and 8505C is in contrast with what observed at mRNA levels. However, this could be ascribed to the general rule of the existence of several levels of regulation between transcript and protein product [38].

As a further mechanistic step, AZD5069 was tested for its ability to revert the basal and CXCL8-induced normal and TC cells migration. Interestingly, AZD5069 significantly inhibited the basal migration in NHT, TPC-1, BCPAP and 8303C but not in 8505C TC cells. Furthermore, the CXCL8-induced TC cell migration was significantly blunted by AZD5069 in NHT and all TC cells types except TPC-1.

Taken together the above data would suggest that treatment with AZD5069 could, at least in part, counteract the CXCL8-induced tumor cell trafficiking not only by selectively blocking the migratory CXCR2 receptor but, at least in some of the here tested thyroid cancer cell types also by down-regulating it.

The observed lack of effect of AZD5069 on 8505C basal migration and on CXCL8-induced migration in TPC-1, could be explained by the observation that the 8505C cells were characterized by the highest basal migration, whereas the TPC-1 cells displayed the most robust migratory response towards CXCL8. The data show that the inhibitory actions of AZD5069 affect cell migration, albeit slightly differently under the concentrations tested in those cancer cells showing a more pronounced migration.

The results of the present study could be of potential clinical relevance. Indeed, the development of immunotherapy strategies, has in recent years opened a novel line of research focused upon the targeting of CXC chemokine/chemokine receptor axis within the tumor microenvironment. In particular, increasing attention has focused on selective CXCR2 antagonism as a therapeutic strategy for cancer and inflammatory diseases [15, 17, 39, 40]. Interference with CXCR2/CXCL8 binding using AZD5069 has recently been proposed as a measure to limit the pro-tumorigenic activity of TANs, by reducing their migration into peritumoral region in vivo [17].

Along these lines, the formation of neutrophil extracellular trap (NETs) resulting in oxidative mitochondrial metabolism changes in TANs represents an additional module regulating pro-tumour activities in anaplastic TC [41]. TANs are associated with poor clinical outcomes in several types of cancer [42–45]. CXCL8 chemoattracts TANs and induces extrusion of NETs, which shield tumor cells that ultimately limit the anti-PD-1 immune response to cancer [46]. The selective anti-CXCR2 blockade using AZD5069 was first reported to selectively block neutrophilic inflammatory pathways in sputum neutrophils derived from COPD patients [47, 48]. In a recent study was demonstrated that the CXCR2 receptor plays a role in the formation of NETs involved in cancer development, thus targeting CXCR2 axis could inhibit tumor progression [49].

In addition, while CXCR1 exclusively binds IL-8, CXCR2 is more promiscuous through binding other potent CXC chemokines (CXCL1-8) [50]. These chemokines are produced by cancer cells themselves and are known to exert pivotal autocrine and paracrine functions that correlate with cancer progression [51], thus justifying the therapeutic targeting of CXCR2 receptors to harness the anti-tumorigenic potential in many cancer types.

In this regard, selective CXCR2 antagonists have been developed (eg AZD5069) with > 100-fold selectivity over the CXCR1 receptors that have been clinically trialled and reported to be well tolerated without adversely affecting neutrophil-mediated host immunity in humans [15].

As far as TC is concerned, the only previous study was performed by using Reparixin, a dual CXCR1/CXCR2 inhibitor [22]. Reparixin was demonstrated to reduce cell viability and stemness of thyroid cancer cells, suggesting that the inhibition of the CXCL8 signaling pathway by either CXC receptor antagonism (i.e., Reparixin) as well as by molecules inhibiting CXCL8 secretion or activity might represent a new strategy for thyroid cancer therapy [22, 31–33, 52]. In addition, a recent study showed that the inhibition of CXCR2 in advanced TC improves the therapeutic effects of MAPK-inhibitors [53].

Notably, recent studies have tested the immunotherapeutic potential of anti-CXCR2 blockade using AZD5069 alone or in combination in many human cancers including; head and neck (NCT02499328), pancreatic (NCT02583477), prostate (NCT03177187), liver [54] and herein, the thyroid.

The present in vitro study evaluated whether a therapeutic strategy aimed at reducing the CXCL8/CXCR2 interplay could counteract TC progression. The results showed that pharmacological blockade of CXCR2 using AZD5069 led to a significant reduction of TC cells migration. However, it should be noted that the reported results herein were obtained in vitro thus requiring further in vivo validation.

Taken together, these results will provide novel insights on the immunotherapeutic potential of this CXC chemokine-chemokine receptor axis in thyroid cancer, which at present remains elusive. Further characterization of the therapeutic significance gained from the selective CXCR2 targeting of TC cells reported herein will contribute to the mechanistic understanding of developing a personalized immunotherapeutic strategy for patients with refractory TC.

## Supplementary Information

Below is the link to the electronic supplementary material.

Supplementary file1 (DOCX 472 KB)

## Data Availability

Data will be available upon request to corresponding author.

## Abbreviations

TC: Thyroid cancer
TSH: Thyroid-stimulating hormone
TM: Tumor microenvironment
TCM: Thyroid cancer microenvironment
CXCL8: Interleukin-8
CXCR1: C-X-C motif chemokine receptor 1
CXCR2: C-X-C motif chemokine receptor
TANs: Tumor-associated neutrophils
mAb: Monoclonal antibody
DMEM: Dulbecco’s Modified Eagle Medium
FBS: Fetal bovine serum
MEM: Minimum Essential Medium
NHT: Normal human thyroid cells
TNF-α: Tumor-necrosis-factor-α
ANOVA: Analysis of variance
NS: Not significant
NETs: Neutrophil extracellular traps
