# Supplementary material for: Selective anti-CXCR2 receptor blockade by AZD5069 inhibits CXCL8-mediated pro-tumorigenic activity in human thyroid cancer cells in vitro
Source: J Endocrinol Invest. 2024 Jun 20;48(1):53–65. doi: 10.1007/s40618-024-02410-6 (PMC11729135; doi:10.1007/s40618-024-02410-6)
Supplement: Supplementary file 1 — Supplementary file1 (DOCX 472 KB) [file 40618_2024_2410_MOESM1_ESM.docx]

*Supplemental materials*

**Selective anti-CXCR2 receptor blockade by AZD5069 inhibits CXCL8-mediated pro-tumorigenic activity in human thyroid cancer cells *in vitro***.

Francesca Coperchini^1^, Alessia Greco^1^, Elena Petrosino^1^, Laura Croce^1,2^, Marsida Teliti^1,2^, Nicoletta Marchesi^3^, Alessia Pascale^3^, Benedetto Calì^4^, Flavia Magri^1,2^, Mohib Uddin^5^, Mario Rotondi^1,2^

^1^ Department of Internal Medicine and Therapeutics, University of Pavia, 27100, Italy;

^2^ Istituti Clinici Scientifici Maugeri IRCCS, Unit of Endocrinology and Metabolism, Laboratory for Endocrine Disruptors, 27100, Pavia, Italy

^3^ Department of Drug Sciences, Unit of Pharmacology, University of Pavia, 27100 Pavia, Italy.

^4^ Istituti Clinici Scientifici Maugeri IRCCS, Department of General and Minimally Invasive Surgery, Pavia (PV), 27100, Italy

^5^ AstraZeneca Gothenburg, Biopharmaceuticals R&D, Mӧlndal, Sweden.

***Key words:*** AZD5069, Thyroid cancer, CXCL8, CXCR2 receptor

*Corresponding Author:*

Prof. Mario Rotondi, M.D., Ph.D.

Unit of Endocrinology and Metabolism, Istituti Clinici Scientifici Maugeri IRCCS

Department of Internal Medicine and Therapeutics, University of Pavia, Italy

Via S. Maugeri 4, I-27100, Pavia, Italy

Fax: +39-0382-592692

e-mail: [mario.rotondi@icsmaugeri.it](mailto:mario.rotondi@icsmaugeri.it)


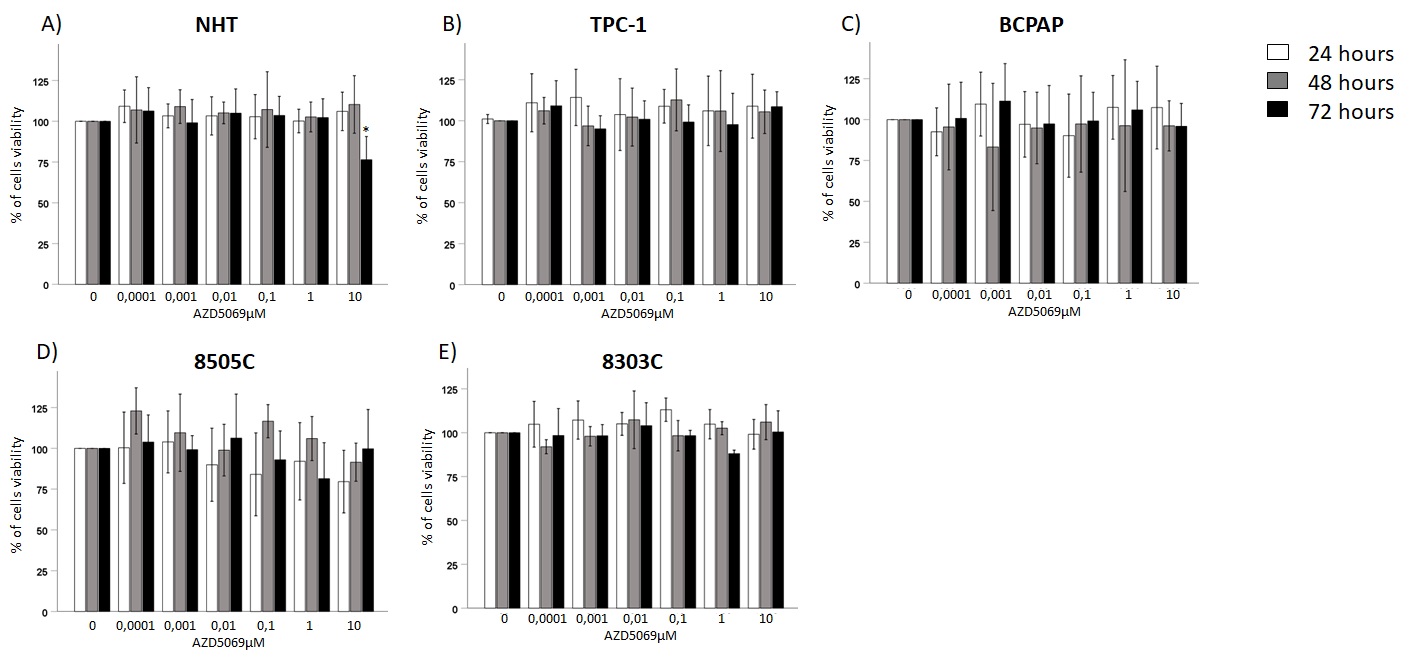


**Figure 1. Effects of AZD5069 treatment on normal and tumor thyroid cell viability:** Treatment of various thyroid cancer cell lines with increasing concentrations of AZD5069 in indicated that the viability of: Panel A) NHT cells were unaffected after 24 hr (ANOVA: F= 0,866 p=0,513, white bars) and 48 hr (ANOVA: F= 0,487 p=0,819, grey bars). whilst 10 µM reduced viability after 72 hr (ANOVA: F= 5,331 p<0,001, black bars). Panel B) TPC-1 cells were unaffected after 24 hr (ANOVA F= 0,047 p=0,820, white bars), 48 hr (ANOVA: F= 1,215 p=0,311, grey bars), and 72 hr (F= 2,011 p= 0,078, black bars). Panel C) BCPAP cells were unaffected after 24 hr (ANOVA: F= 1,293 p=0,274, white bars), 48 (ANOVA: F= 2,117 p=0,064, grey bars) and 72 hr (ANOVA: F= 1,361 p= 0,253, black bars). Panel D) 8505c cells were unaffected after 24 hr (ANOVA F= 1,234 p=0,306, white bars), 48 hr (ANOVA: F=0,162 p=0,986, grey bars) and 72 hr (ANOVA: F= 1,326 p= 0,256, black bars). Panel E) 8303C cells were unaffected after 24 hr (ANOVA: F=0.883, p=0,532, white bars), 48 hr (F=1,144,p=0,388, grey bars) and 72 hr (F=0,829, p=0,567, black bars).

**post hoc by Bonferroni p<0.05*


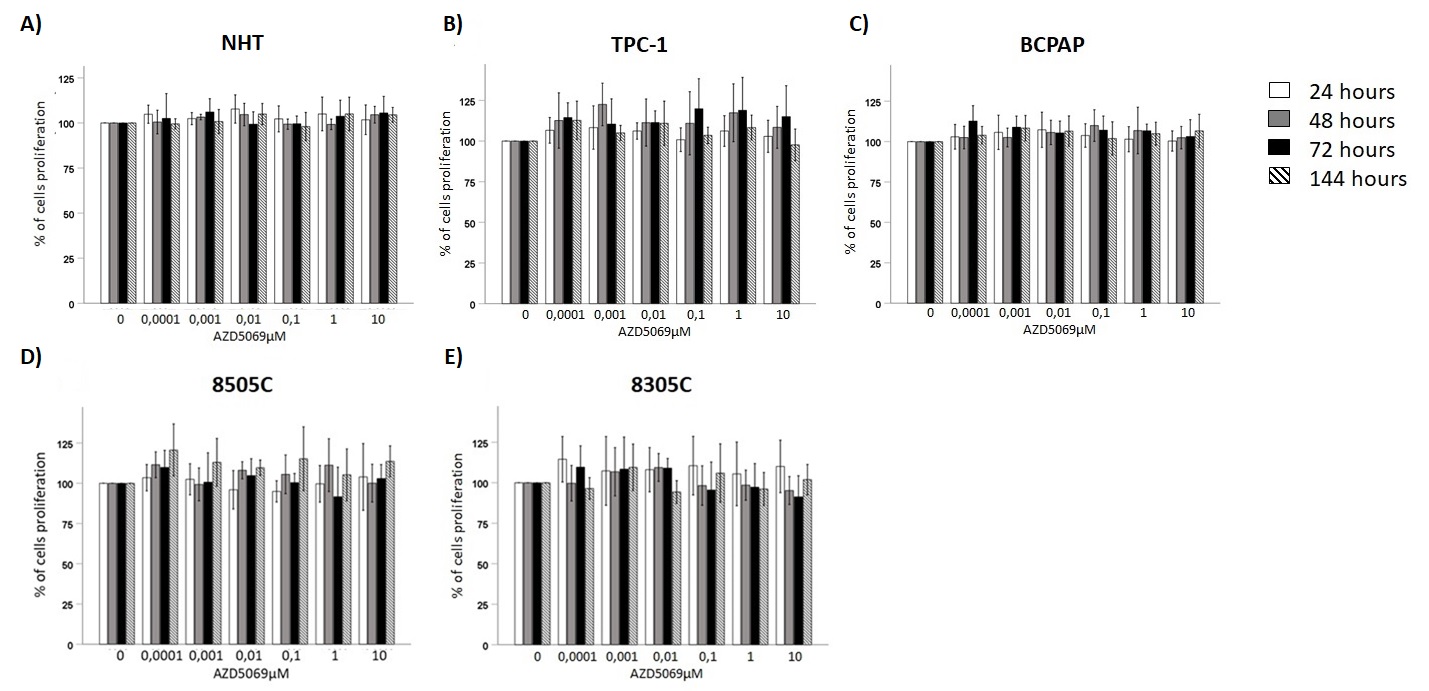


**Figure 2. Effect of AZD5069 on NHT and thyroid cancer cell proliferation.** Treatment of various NHT and thyroid cancer cell lines with increasing concentrations of AZD5069 [100 pM-10 µM] throughout the time-course did not modify the proliferation in Panel A) NHT (ANOVAs: 24 hr F= 0,613 p= 0,717, white bars; 48 hr F= 1,354 p=0,278, grey bars 72 hr F= 0,477 p=0,818, black bars; 144 hr F= 0.953 p=0,479, striped bars); Panel B) TPC-1 cells (ANOVAs: 24 hr F= 0,715 p= 0,639, white bars; 48 hr F= 1,859 p=0,107, grey bars 72 hr F= 1,994 p=0,077, black bars; 144 hr F= 1,797 p=0,119, striped bars); Panel C) BCPAP cells (ANOVAs: 24 hr F= 0,822, p= 0,559, white bars; 48 hr F= 0,994 p=0,436, grey bars, 72 hr F= 1,582 p=0,165, black bars; 144 hr F= 0,761 p=0,604, striped bars). Panel D) 8505C cells (ANOVAs: 24 hr F= 0,783 p= 0,587, white bars; 48 hr F= 2,025 p=0,073, grey bars, 72 hr F= 1,759 p=0,127, black bars; 144 hr F= 1,892 p=0,101, striped bars). Panel E) 8305C cells (ANOVAs: 24 hr F= 1,174 p= 0,331, white bars; 48 hr F= 1,638 p=0,161, grey bars, 72 hr F= 1,795 p=0,124, black bars; 144 hr F= 1,519 p=0,192, striped bars).

**post hoc by Bonferroni p<0.05*


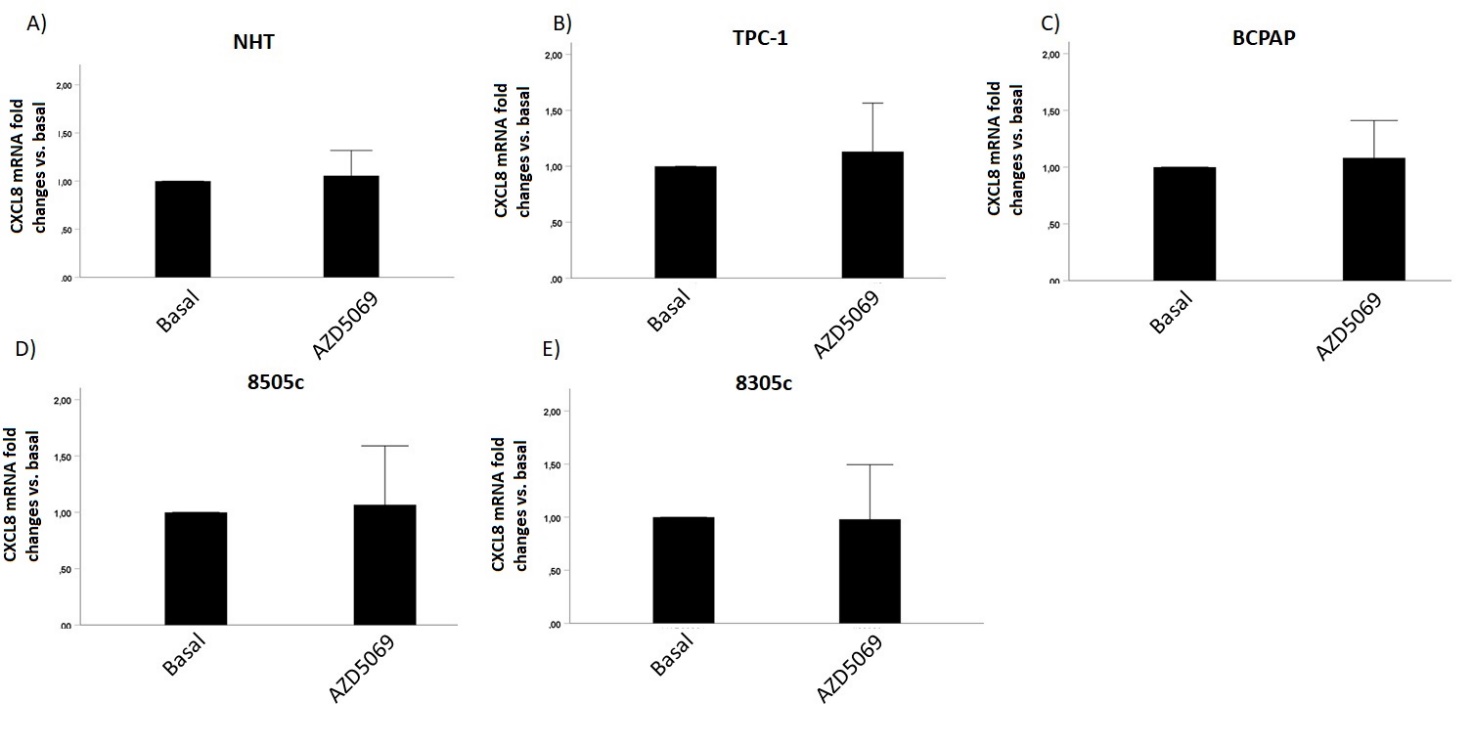


**Figure 3. Effect of AZD5069 on CXCL8 mRNA levels in normal and tumor thyroid cells**

Treatment with AZD5069 [1 µM did not significantly (*Student’s t-test*) modify mRNA levels of the CXCL8 ligand expressed by the various thyroid cancer cell lines tested: Panel A) NHT cells; Panel B) TPC-1 cells; Panel C) BCPAP cells; Panel D) 8505C cells; Panel E) 8305C cells.

**Student t-test p<0.05*
